# The earliest direct evidence of frogs in wet tropical forests from Cretaceous Burmese amber

**DOI:** 10.1038/s41598-018-26848-w

**Published:** 2018-06-14

**Authors:** Lida Xing, Edward L. Stanley, Ming Bai, David C. Blackburn

**Affiliations:** 1 State Key Laboratory of Biogeology and Environmental Geology, China University of Geosciences, Beijing, 100083 China; 2 School of the Earth Sciences and Resources, China University of Geosciences, Beijing, 100083 China; 3 Florida Museum of Natural History, University of Florida, Gainesville, 32611 Florida USA; 4 Key Laboratory of Zoological Systematics and Evolution, Institute of Zoology, Chinese Academy of Sciences, Beijing, 100101 China

## Abstract

Frogs are a familiar and diverse component of tropical forests around the world. Yet there is little direct evidence from the fossil record for the antiquity of this association. We describe four fossil frog specimens from mid-Cretaceous (~99 mya) amber deposits from Kachin State, Myanmar for which the associated fauna provides rich paleoenvironmental context. Microcomputed tomographic analysis provides detailed three-dimensional anatomy for these small frogs, which is generally unavailable for articulated anurans in the Mesozoic. These crown-group anuran specimens provide the earliest direct evidence for anurans in a wet tropical forest. Based on a distinct combination of skeletal characters, at least one specimen has clear similarities to living alytoid frogs as well as several Mesozoic taxa known from the Jehol Biota in China. Whereas many Mesozoic frogs are from seasonal and mesic paleoenvironments, these fossils provide the earliest direct evidence of anurans in wet tropical forests.

## Introduction

Frogs originated >200 mya, but the fossil record for Mesozoic anurans is relatively depauperate^1^. Most extant families of anurans likely originated by the end of the Paleogene with major geographically circumscribed clades originating even earlier in the Cretaceous^2^. The living diversity of anurans (>6,900 species)^3^ encompasses a range of morphotypes associated with different microhabitat specializations and various reproductive and locomotor modes^4–7^. Present-day tropical forests harbor an extraordinary diversity of frogs in both the number of species and axes of phenotypic variation such as reproductive modes^8–10^. However, there is little direct evidence from the fossil record of this association largely due to the limited paleoecological context for most Mesozoic anurans.

Many species in the diverse clade comprising ‘modern frogs’—the Neobatrachia—are found in tropical forests. However, among other extant frogs not included within the Neobatrachia, only the Pipidae, Megophryidae, and the genus *Barbourula* (Bombinatoridae) are associated with tropical forests. Time-calibrated molecular phylogenies indicate that both *Barbourula* and megophryid frogs, and thus likely their association with tropical forests, originated in the mid-Paleogene^2,11^. Based on the ecology and distribution of extant pipid frogs, this clade of largely aquatic species might have been associated with tropical freshwater habitats in southern Gondwana during the Mesozoic, though the paleoecological context for many relevant fossils remains limited. Recent phylogenetic analysis suggests that all extant families and subfamilies containing arboreal species originated after the Cretaceous^2^, suggesting that new ecological opportunities shaped anuran diversification as forests rebounded after the massive vegetation loss at the K–Pg extinction event^12^.

The amber deposits of northern Myanmar provide a unique record of a forest ecosystem during the Upper Albian (~99 mya^13,14^). Both plants, including mosses and bamboo-like monocots^15,16^, and invertebrates, including pisaurid spiders, onycophorans, dyspnoid harvestman, and coccoid scale insects^13,17,18^, preserved in Burmese amber provide evidence that this was a humid, warm, tropical forest ecosystem that contained at least some freshwater habitats. The presence of ammonites and marine ostracods suggest that some of the amber-bearing forests occurred near the shore of a marine environment^19^. Vertebrates have recently been reported from these deposits, including well-preserved three-dimensional anatomy of skeletons and feathers^20–22^. These amber deposits represent an excellent opportunity for discovering three-dimensionally preserved small vertebrates with a rich associated paleoecological context.

We report the first specimens of frogs preserved in amber from northern Myanmar. These are the oldest records of frogs preserved in amber, with the only two previous reports from Cenozoic amber deposits of the Dominican Republic^23,24^. These Burmese fossils provide the earliest direct evidence of anurans in a wet tropical forest ecosystem.

## Results

### Systematic Paleontology

Order Anura Fischer von Waldheim, 1813.

? Alytoidea Fitzinger, 1843.

Family undetermined.

Genus *Electrorana* gen. nov. LSID, urn:lsid:zoobank.org:act: C4047BE5-2894-4B3D-8833-6DE99935D0B6.

Type species *Electrorana limoae* sp. nov.; by present designation LSID, urn:lsid:zoobank.org:act:676DC783-6773-46B4-A018-F854C5AC421C

### Etymology

*Electrorana* is feminine and derives from the Latin *electrum* (amber) and *rana* (frog). The specific epithet, *limoae*, is a matronym in the genitive singular for Mrs. Mo Li, who purchased and provided these specimens for study.

### Material

**Figure 1:**
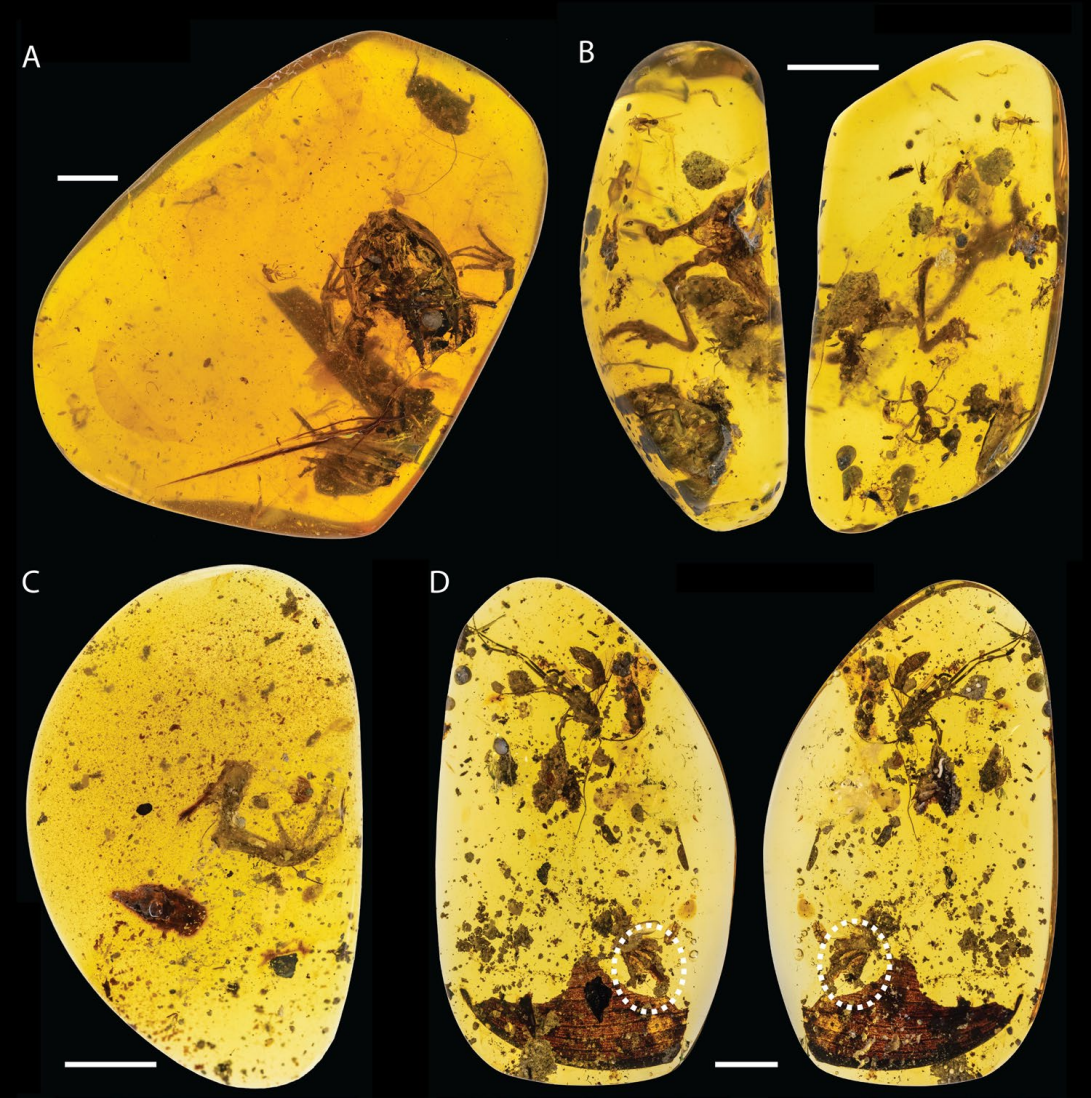
Photograph images of four fossil frog specimens referred to *Electrorana*, including the holotype (**A**; DIP-L-0826) and three additional specimens (**B**: DIP-V-16119; **C**: DIP-V-16127; **D**: DIP-V-16121). Specimens in (**B**) and (**D**) are presented with two views of the amber specimen and the oval in (**D**) indicates the presence of the anuran specimen. Scale bars equal 5 mm.

**Figure 2:**
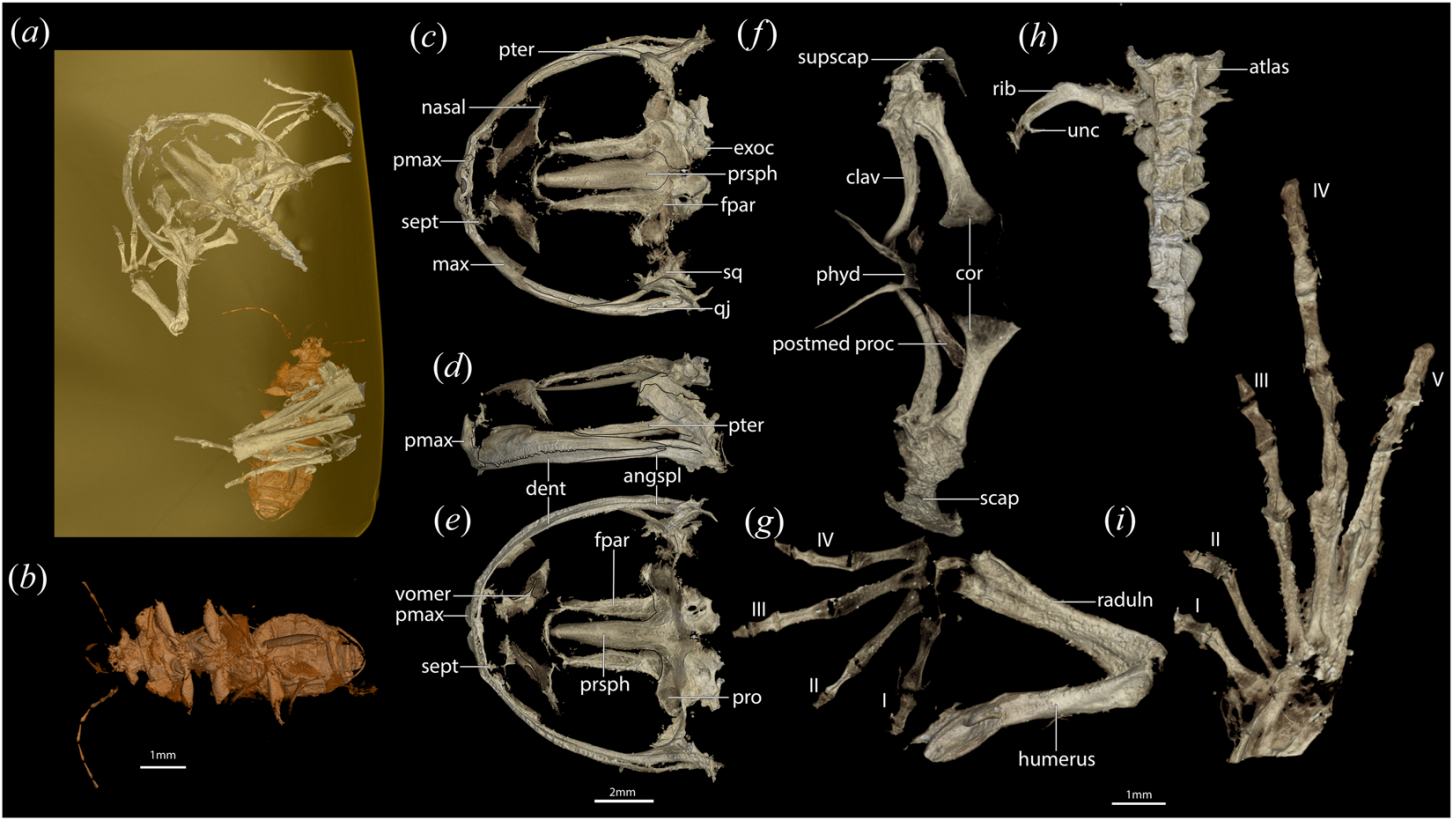
Holotype (**a**) of *Electrorana limoae* (DIP-L-0826) as visualized via microCT scanning. (**b**) Unidentified coleopteran embedded in amber with holotype. Skull in dorsal (**c**), left lateral (**d**), and ventral (**e**) views. Ventral views of pectoral girdle (**f**), left hand (**g**), vertebral column (**h**), and left foot (**i**). Abbreviations of anatomical terms are as follows: clav – clavicle; cor – coracoid; dent – dentary; exoc – exoccipital; fpar – frontoparietal; max – maxilla; phyd – parahyoid; pmax – premaxilla; postmed proc – posteromedial process of hyoid; pro – prootic; prsph – parasphenoid; pter – pterygoid; qj – quadratojugal; raduln – radioulna; scap – scapula; sept – septomaxilla; sq – squamosal; supscap – suprascapula; unc – uncinate process. The number of each digit is indicated with roman numerals. Scale bar for (**c**–**e**), 2 mm; scale bar for (**b**,**f**–**i**) 1 mm.

**Figure 3:**
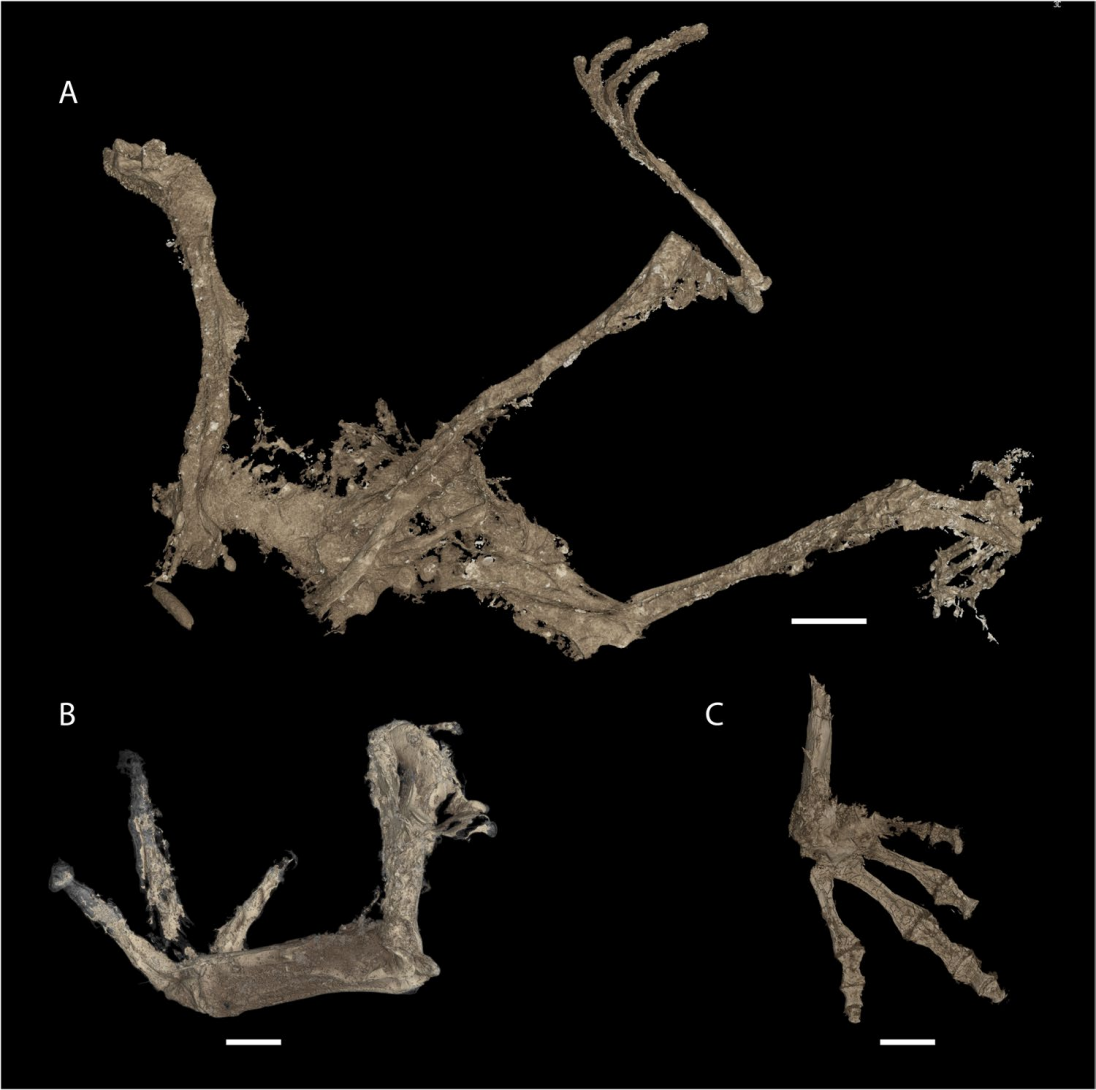
Three fossil frog specimens referred to *Electrorana* visualized via microCT scanning. (**A**) DIP-V-16119, (**B**) DIP-V-16127, (**C**) DIP-V-16121. Scale bars equal 2 mm.

Four fossil frog specimens (DIP-V-16119, DIP-V-16121, DIP-V-16127, and DIP-L-0826) are catalogued in the Dexu Institute of Palaeontology (DIP), Chaozhou, China (Figs 1, 2 and 3). A 3D-printed replica of DIP-L-0826 is deposited at the Florida Museum of Natural History (FLMNH VP-312847; Supplemental Fig. 1). The holotype (DIP-L-0826) is a partial skeleton embedded within a block of amber (Figs 1 and 2) that also contains an unidentified beetle (Coleoptera; D. Grimaldi, pers. comm.). Three other specimens preserve either an incomplete anuran forelimb (DIP-V-16121, 16127) or the general body shape of a frog that x-ray computed tomography reveals contains no skeletal material within (DIP-V-16119).

### Type locality and horizon

These amber-preserved specimens were acquired in the area of Angbamo in Kachin Province of northern Myanmar in August 2015. Burmese amber derives from late Albian–Cenomanian deposits (approx. 105–95 mya^25^). Dating of zircons from the volcaniclastic matrix in these deposits provides an age of 98.8 ± 0.6 million years^14^.

### Diagnosis

*Electrorana* differs from extant and extinct anurans by the combination of a premaxilla with a prominent and bifurcated alary process, a V-shaped parahyoid bone, and free ribs (preserved on Presacral vertebra II), as well as the absence of a palatine.

### Description

The holotype is a partial skeleton of a small frog (~22 mm snout–vent length) preserving a skull, a nearly complete left forelimb, pectoral girdle, partial right forelimb, a partial preaxial vertebral column, and a partial left hindlimb. The neurocranium has been flattened such that the frontoparietals and sphenethmoid rest alongside the parasphenoid. The sacrum, urostyle, and pelvis are not preserved, nor are the dorsal components of the vertebrae (Supplemental Materials). Ossified sternal elements and posteromedial processes of the hyoid might have been displaced and not preserved.

The skull is approximately as wide as long (~9 mm in length and width) and relatively flat in lateral view. In dorsal view, the jaw joint is approximately even with the occiput. The dermal elements of the skull are not sculptured. The triangular nasals are widely spaced, form the anterior border of the orbit, have a weakly concave anterolateral margin, and taper posteriorly; the nasal does not bear a rostral process or articulate with the maxilla. The frontoparietals are paired and unfused, bordering a large fontanelle. The frontoparietal does not have a supraorbital flange or posterolateral process. The squamosal is present but difficult to discern due to pyritization of overlying soft tissues. The premaxilla is dentigerous (~10–12 teeth) and bears a stout, forked alary process extending dorsally. The palatine process of the premaxilla is weakly developed. A small triradiate septomaxilla is posterolateral to the alary process of the premaxilla. The stout maxilla articulates with the premaxilla, but is relatively shorter, and bears a deep facial process that tapers posteriorly along the orbit. The maxilla bears teeth along three-fourths of its length (~25 teeth). Posteriorly, the maxilla has an overlapping articulation with a slender quadratojugal. The vomer bears pre- and post-choanal processes with a dentigerous process (5–6 teeth) medial to the prechoanal process. There is no palatine. The sphenethmoid is present as a thin posteriorly directed C-shaped mineralization extending across the midline at the anterior margin of the parasphenoid; an ossified nasal septum is not present. The pterygoid is thin and tri-radiate, abutting the maxilla, lateral margin the parasphenoid ala, and the pyritized mass containing the squamosal and quadrate. The parasphenoid is triradiate, tapering anteriorly and nearly reaching the vomers, with alae extending across the ventral surface of the prootics and exoccipitals, which are not co-ossified. The occipital condyles are widely space.

The lower jaw does not bear teeth. Separate elements of the lower jaw, including the mentomeckelians, are difficult to discern but it appears to be comprised of a dentary and angulosplenial. Medial and anterior to the clavicles, there is a thin V-shaped parahyoid bone. Posteromedial processes of the hyoid are present posterolateral to the parahyoid and dorsal to the coracoids.

The pectoral girdle was likely arciferal, as suggested by the anteriorly curved clavicles. The clavicle tapers medially and is approximately twice the length of the stout scapula. The coracoid is approximately the same length as the clavicle, and is weakly expanded medially. Both the coracoid and clavicle articulate with the scapula. The clavicles are widely spaced, and there are no ossified sternal elements. There may be a cleithrum present but it is not clearly demarcated from the suprascapula. The humerus and fused radiulna are approximately equal in length. The metacarpals are relatively straight and lack processes along their lateral margins; metacarpal III is the longest. The carpals are not fully ossified and thus difficult to discern. The phalangeal formula of the manus is 2-2-3-3, and a prepollex is absent. The terminal phalanges of the manus are subtriangular and weakly expanded at their distal tip.

The vertebral column is incomplete, but portions of presacral vertebrae I–VII are preserved. Neural arches are not preserved and a transverse process is only preserved on Presacral II. The atlas does not bear transverse processes, and the atlantal cotyles are widely spaced and not contiguous. A free rib is preserved in articulation with the right transverse process of Presacral II, possibly with a posteriorly directed uncinate process. The centra of the presacral vertebrae are difficult to discern.

The hindlimb is incompletely preserved, but longer than the forelimb. Neither the femur nor the fused tibiofibula is preserved in its entirety. The tarsals are not fully ossified. An enlarged prehallux is absent. The metatarsals are all similar in length. The phalangeal formula of the pes is 2-2-3-4-3.

### Remarks

The holotype of *Electrorana* is likely not an adult. This is based on the absence of the columella and incomplete ossification of the carpals, prootic-exocciptal, and sphenethmoid. These often form or fully ossify after metamorphosis^26^. For example, in *Bombina* ossification of the columella may not happen until late in ontogeny (>2 years of age)^27^.

### Phylogenetic analyses

Because previous phylogenetic analyses of Mesozoic anurans have produced conflicting results for the relationships of some taxa, we included *Electrorana* in three different recent matrices that differ in both their characters and taxa. Analysis of the 72 characters and 27 taxa (including *Electrorana*) based on the matrix from Báez^28^ resulted in nine equally parsimonious trees (score = 260); the matrix contains 20% missing data, including 41 of 72 characters for *Electrorana*. Analysis of the 97 characters and 52 taxa (including *Electrorana*) based on the matrix from Gao & Chen^29^ resulted in 598,547 equally parsimonious trees (score = 381); the matrix contains 28% missing data, including 47 of 97 characters for *Electrorana*. Analysis of the 66 characters and 27 taxa (including *Electrorana*) based on the matrix from Henrici *et al*.^30^ resulted in four equally parsimonious trees (score = 243); the matrix contains 20% missing data, including 36 of 66 characters for *Electrorana*.

**Figure 4:**
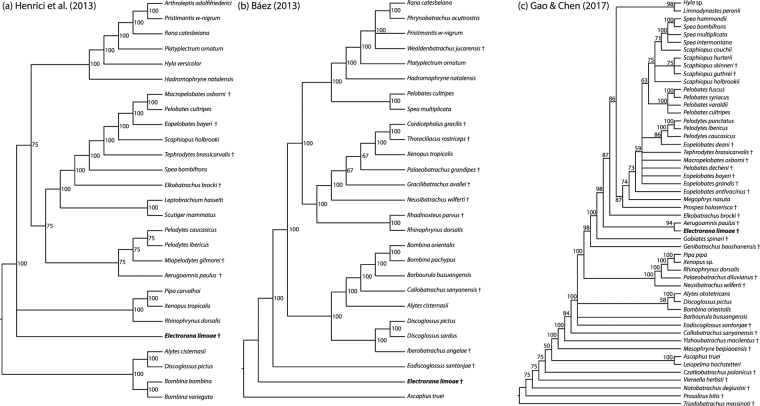
Majority-rule consensus trees representing phylogenetic analyses using parsimony of matrices from (**a**) Henrici *et al*.^30^, (**b**) Báez^28^, and (**c**) Gao & Chen^29^ including *Electrorana* from the Cretaceous of Myanmar. Numbers adjacent to nodes represent the percentage of equally parsimonious trees containing that node. The phylogeny based on Henrici *et al*.^30^ is rooted using the extant alytoid clade, that based on Báez^28^ is rooted with *Ascaphus*, and that based on Gao & Chen^29^ is rooted with *Triadobatrachus*. Extinct taxa are indicated with a dagger, and *Electrorana* is indicated in boldface.

The topology based on the matrix from Gao & Chen^29^ clearly indicates that *Electrorana* is a crown-group anuran and supports an affinity with the extinct taxon *Aerugoamnis* from the Early Eocene Green River Formation of Wyoming (Fig. 4). However, analyses based on the matrices of Henrici *et al*.^30^ and Báez^28^ suggest that *Electrorana* may be an earlier diverging lineage of crown-group anurans. Notably, analysis of the matrix of Henrici *et al*.^30^ which was used in the recognition and description of *Aerugoamnis*, does not suggest a close relationship between *Electrorana* and *Aerugoamnis*. Because of both the large amount of missing data in these matrices, as well as the relatively small number of characters (ranging from 66 to 97 characters), the phylogenetic affinities of *Electrorana* remain uncertain. However, based on the phylogenetic analysis, we are confident that *Electrorana* is not within crown-group Acosmanura, which includes Anomocoela and Neobatrachia. Further, among extant taxa, *Electrorana* has strong anatomical similarities to extant alytoids (formerly referred to as Discoglossoidea^31^).

## Discussion

### Evolutionary relationships

*Electrorana* bears strong similarities to extant taxa that form the clade Alytoidea, including Bombinatoridae and Alytidae. These are the only extant taxa exhibiting the unique combination of a V-shaped parahyoid bone, free ribs, and lacking a palatine. Among extinct Mesozoic anurans, *Electrorana* is similar to anurans from the older Jehol Biota. These are recognized as either the single genus *Liaobatrachus*^32^ or a collection of crown-group anurans with affinities to extant Alytoidea^33^. Other taxa with similarities to *Electrorana* include *Eodiscoglossus* from the Jurassic and Lower Cretaceous of Europe^1,34,35^ and two taxa from the Lower Cretaceous of Japan^36^. However, phylogenetic analyses conducted by different authors result in conflicting patterns of relationships for these Mesozoic taxa, including whether these are all within crown-group Anura^28,32–36^. The incompleteness of the holotype of *Electrorana*, especially the lack of a sacrum, urostyle, and pelvis, complicates determining its affinities among these Mesozoic anurans. It is diagnosable as a member of crown-group Anura by the presence of a V-shaped parahyoid which is not known in stem anurans.

### Comparisons

Among extant anurans, *Electrorana* is most similar to taxa in the families Alytidae and Bombinatoridae, which together comprise the Alytoidea. While today representing only five genera, many extinct taxa extending into the late Jurassic have been referred to this clade^34,37^. In comparison to *Ascaphus* (Ascaphidae) and *Leiopelma* (Leiopelmatidae), which comprise the earliest diverging lineage of extant anurans, *Electrorana* has more robust premaxillae that are approximately as tall as wide in anterior view. In both *Ascaphus* and *Leiopelma*, the premaxillae are wide with a narrow alary process. The alary process of the premaxilla in *Electrorana* is robust, comprising approximately half the length of the bone, which is most similar to the condition of *Alytes* (Alytidae) and *Bombina* (Bombinatoridae). The alary process is also forked dorsally, a condition similar to that recently reported for *Genibatrachus* by Gao & Chen^29^. The nasals of *Electrorana* are widely spaced and slender, similar to *Ascaphus* and *Bombina* (Bombinatoridae) and believed to be the ancestral condition for anurans^38^. The frontoparietals of *Electrorana* are widely spaced as in *Ascaphus*, *Leiopelma*, and *Alytes*, though unlike *Bombina* and *Barbourula* (Bombinatoridae) in which the fontanelle is partially or fully covered. The dentigerous vomer of *Electrorana* is more complicated in shape than the simple vomer found in *Ascaphus* (which does not have a postchoanal ramus of the vomer^38^), but similar in structure to the vomer of other anurans including *Leiopelma*, *Alytes*, *Bombina*, or *Pelodytes* (Pelodytidae). Unlike these other taxa, the vomer of *Electrorana* is relatively small and displaced anteriorly, which would suggest a relatively anterior location for the choana; however, this may be a result of either the holotype not being an adult specimen or being displaced postmortem. While thin and small, the sphenethmoid of *Electrorana* does appear to be contiguous across the midline, unlike the condition found in *Ascaphus* and *Leiopelma* in which it is generally poorly ossified^38^. The sphenethmoid is clearly not as robust as seen in *Alytes*, *Discoglossus*, *Bombina*, and many other anurans, but this is likely due to the holotype of *Electorana* not being an adult specimen. The pterygoids lack the enlarged ventrolaterally projecting flange found in *Barbourula*.

There is a thin V-shaped parahyoid bone, as found in *Alytes*, *Discoglossus*, and *Pelodytes*^38^, but there are no plate-like parahyoid bones as found in *Bombina*, *Barbourula*, or *Rhinophrynus* (Rhinophrynidae). A similar V-shaped parahyoid is found in the various anuran taxa referred to as *Liaobatrachus*^32,34^, including *Callobatrachus*, *Mesophryne*, *Dalianbatrachus*, and *Yizhoubatrachus*^33^. Unlike the condition in *Pelodytes*, the parahyoid bone does not appear ‘x-shaped’ with posterolateral processes that approach the bony posteromedial processes of the hyoid.

The one preserved rib of *Electorana* is relatively longer than seen in *Alytes* and *Bombina*. In *Bombina*, the ribs of Presacral II and III can bear a similar posteriorly directed uncinate process to what appears to be present in *Electrorana*. Unlike taxa in the Megophryidae, Pelobatidae, Pelodytidae, and many neobatrachian frogs, *Electrorana* does not exhibit ossified sternal elements along the midline.

### Paleoenvironment

The paleoecological context for most Mesozoic anurans is either unknown or not described as being a tropical forest. The paleoecology of the Jehol Biota in China (~130–122 mya^39^) is probably the best documented for a Cretaceous faunal assemblage that contains anurans. It is characterized as having seasonal and mesic environments^40^ and possibly open forested habitats^41^. The Lower Cretaceous Crato Formation of Brazil is another example with anuran fossils coming from lacustrine deposits that were also likely formed in a semiarid environment^42^. Other examples of paleoenvironments include *Notobatrachus* from the Jurassic La Matilde Formation of Argentina^43^ and the late Cretaceous *Paralatonia* from the Haţeg Basin of Romania^44^, both of which were associated with low-energy freshwater environments including floodplains and swamps. The earlier Triassic stem-anuran *Triadobatrachus* likely lived in semi-arid, near-shore riparian forests near floodplains^45,46^. Transitions to more arid paleoenvironments have even been cited as a possible driver of morphological evolution in the earliest anurans^47^. *Electrorana* from the mid-late Cretaceous amber deposits in Myanmar provides the oldest definitive association of anurans with tropical forests and suggests that lineages today associated with temperate regions may have occupied a larger diversity of habitats in the past.

## Methods

We performed high-resolution x-ray computed tomography (CT-scanning) at the University of Florida’s Nanoscale Research Facility. We used a Phoenix v|tome|x M (GE Measurement & Control, Boston, USA) scanner with a 180 kv x-ray tube with a diamond-tungsten target and with the following settings: 75 kV, 150 mA, a 0.5 second detector time, averaging of three images per rotation and a voxel resolution of 8.6–10.7 µm. Raw 2D x-ray data were processed using the datos|x software v. 2.3 with post-processing, analyses (including segmentation), and visualization conducted using VG StudioMax v. 3.1 (Volume Graphics, Heidelberg, Germany).

A replica of DIP-L-0826 (enlarged by 300%) was created by 3D-printing.stl files that were extracted from the CT volumetric data. The replica was printed using rigid polypropylene materials using an Objet260 Connex2 3D printer (Stratasys, Eden Prairie, USA) based at the University of Florida’s Nanoscale Research Facility.

Morphological comparisons to other anurans were based on CT-scans of extant species also scanned at the University of Florida and fossil specimens housed at the Institute for Vertebrate Paleontology and Paleoanthropology in Beijing, China (see Supplementary Table 1), as well as published descriptions of extinct anurans.

To evaluate potential evolutionary relationships of *Electrorana*, we coded this new taxon into three different recent matrices used in phylogenetic analyses of extinct and extant anurans^28–30^. We conducted phylogenetic analyses using parsimony as an optimality criterion in PAUP* v4.0a (build 158)^48^. For the analysis of each matrix, we conducted 10 replicate heuristic searches with starting trees obtained through random stepwise addition. Missing (?) or inapplicable (−) character states were both treated as missing data in the analyses; taxa with multistate characters were treated as uncertainties (rather than polymorphisms). We summarized the results using majority rule consensus and these are presented in Fig. 4.

### Data accessibility

Tomograms (TIFF) and shape files (STL) are available from MorphoSource (http://morphosource.org/). See Supplemental Materials for further details.

## Electronic supplementary material

Supplementary Materials

Supplemental Video 1

Supplemental Video 2

Supplemental Video 3

## References

[1] Roček Z. In *Amphibian Biology, volume 4. Paleontology*. (eds Heatwole, H. & Carroll, R. L.) 1295–1331 (Surrey Beatty & Sons, Chipping Norton, 2000).

[2] Feng YJ (2017). Phylogenomics reveals rapid, simultaneous diversification of three major clades of Gondwanan frogs at the Cretaceous–Palaeogene boundary. Proc. Nat. Acad. Sci. USA.

[3] AmphibiaWeb. AmphibiaWeb: Information on amphibian biology and conservation. University of California, Berkeley. Available at amphibiaweb.org. Accessed 2 February 2018.

[4] Haddad CFB, Prado CPA (2005). Reproductive modes in frogs and their unexpected diversity in the Atlantic Coastal Forest of Brazil. BioScience.

[5] Moen DS, Morlon H, Wiens JJ (2016). Testing convergence versus history: convergence dominates phenotypic evolution for over 150 millions years in frogs. Syst. Biol..

[6] Moen, D. S. & Wiens, J. J. Microhabitat and climatic niche change explain patterns of diversification among frog families. *Am. Nat*. **190** (2017). 10.1086/692065 28617640

[7] Portik DM, Blackburn DC (2016). The evolution of reproductive diversity in Afrobatrachia: A phylogenetic comparative analysis of an extensive radiation of African frogs. Evolution.

[8] Wells, K. D. *The ecology and behavior of amphibians*. (University of Chicago Press, Chicago, 2010).

[9] Wiens JJ, Pyron RA, Moen DS (2011). Phylogenetic origins of local-scale diversity patterns and the causes of Amazonian megadiversity. Ecol. Letters.

[10] Zamudio K, Bell RC, Nali RC, Haddad CFB, Prado CPA (2016). Polyandry, predation, and the evolution of frog reproductive modes. Am. Nat..

[11] Blackburn DC, Bickford DP, Diesmos AC, Iskandar DT, Brown RM (2010). An ancient origin for the enigmatic flat-headed frogs (Bombinatoridae: *Barbourula*) from the islands of Southeast Asia. PLoS One.

[12] Vajda V, Raine JI, Hollis CJ (2001). Indication of global deforestation at the Cretaceous- Tertiary boundary by New Zealand fern spike. Science.

[13] Grimaldi DA, Engel MS, Nascimbene PC (2002). Fossiliferous Cretaceous amber from Myanmar (Burma): its rediscovery, biotic diversity, and paleontological significance. Am. Mus. Novit..

[14] Shi G (2012). Age constraint on Burmese amber based on U–Pb dating of zircons. Cret. Res..

[15] Bell NE, York PV (2007). *Vetiplanaxis pyrrhobryoides*, a new fossil moss genus and species from Middle Cretaceous Burmese amber. Bryologist.

[16] Poinar GO (2004). *Programinis burmitis gen. et sp. nov*., and *P. laminatus sp. nov*., Early Cretaceous grass-like monocots in Burmese amber. Aust. Syst. Bot..

[17] Giribet G, Dunlop JA (2005). First identifiable Mesozoic harvestman (Opiliones: Dyspnoi) from the Cretaceous Burmese amber. Proc. R. Soc. B.

[18] Penney D (2004). A new genus and species of Pisauridae (Aranae) in Cretaceous Burmese amber. J. Syst. Palaeontol..

[19] Xing LD (2018). A gigantic marine ostracod (Crustacea: Myodocopa) trapped in mid-Cretaceous Burmese amber. Scientific Reports.

[20] Daza JD, Stanley EL, Wagner P, Bauer AM, Grimaldi DA (2016). Mid-Cretaceous amber fossils illuminate the past diversity of tropical lizards. Sci. Adv..

[21] Xing LD (2016). A feathered dinosaur tail with primitive plumage trapped in Mid-Cretaceous amber. Current Biol..

[22] Xing LD (2017). A mid-Cretaceous enantiornithine (Aves) hatchling preserved in Burmese amber with unusual plumage. Gondwana Res..

[23] Poinar GO, Cannatella DC (1987). An Upper Eocene frog from the Dominican Republic and its implications for Caribbean biogeography. Science.

[24] Anderson SR (2004). Insect meals from a leptodactylid frog (Amphibia: Leptodactyidae [sic]) in Dominican amber (Miocene, 23 ma). Entomol. News.

[25] Cruickshank RD, Ko K (2003). Geology of an amber locality in the Hukawng Valley, Northern Myanmar. J. Asian Sci..

[26] Maglia AM, Pugener LA (1998). Skeletal development and adult osteology of *Bombina orientalis* (Anura: Bombinatoridae). Herpetologica.

[27] Smirnov SV (1989). Postmetamorphic skull development in *Bombina orientalis* (Amphibia, Discoglossidae), with comments on neoteny. Zool. Anz..

[28] Báez A (2013). Anurans from the Early Cretaceous Lagerstätte of Las Hoyos, Spain: new evidence on the Mesozoic diversification of crown-clade Anura. Cret. Res..

[29] Gao KQ, Chen J (2017). A new crown-group frog (Amphibia: Anura) from the Early Cretaceous of northeastern Inner Mongolia, China. Am. Mus. Novit..

[30] Henrici AC, Báez AM, Grande L (2013). *Aerugoamnis paulus*, new genus and new species (Anura: Anomocoela): first reported anuran from the early Eocene (Wasatchian) Fossil Butte Member of the Green River Formation, Wyoming. Ann. Carnegie Mus..

[31] Dubois A (2005). Amphibia Mundi. 1.1. An ergotaxonomy of recent amphibians. Alytes.

[32] Dong L, Roček Z, Wang Y, Jones MEH (2013). Anurans from the Lower Cretaceous Jehol Group of Western Liaoning, China. PLoS ONE.

[33] Gao KQ, Chen S (2004). A new frog (Amphibia: Anura) from the Lower Cretaceous of western Liaoning, China. Cret. Res..

[34] Roček Z (2013). Mesozoic and Tertiary Anura of Laurasia. Palaeobio. Palaeoenv..

[35] Báez AM, Gómez RO (2016). Revision of the skeletal morphology of *Eodiscoglossus santonjae*, an Early Cretaceous frog from northeastern Spain, with comments on its phylogenetic placement. *Foss*. Imprint.

[36] Ikeda T, Ota H, Matsui M (2016). New fossil anurans from the Lower Cretaceous Sasayama Group of Hyogo Prefecture, Western Honshu, Japan. Cret. Res..

[37] Marjanović D, Laurin M (2014). An updated paleontological timetree of lissamphibians, with comments on the anatomy of Jurassic crown-group salamanders (Urodela). Hist. Biol..

[38] Cannatella, D. C. *A phylogeny of primitive frogs (Archaeobatrachians)*. Unpublished Ph.D. thesis, (University of Kansas, 1985).

[39] Chang SC, Zhang H, Renne PR, Fang Y (2009). High-precision ^40^Ar/^39^Ar age for the Jehol biota. Palaeogeogr. Palaeocl..

[40] Liu PJ, Huang JD, Ren D, Zhao YY (2009). Aquatic community succession and environmental changes of late Mesozoic in northern China. Acta Zootaxonom. Sin..

[41] Zhonghe Z (2006). Evolutionary radiation of the Jehol Biota: chronological and ecological perspectives. Geol. J..

[42] Báez AM, Moura GJB, Gómez RO (2009). Anurans from the Lower Cretaceous Crato Formation of northeastern Brazil: implications for the early divergence of neobatrachians. Cret. Res..

[43] Báez AM, Nicoli L (2004). A new look at an old frog: the Jurassic *Notobatrachus* Reig from Patagonia. Ameghiniana.

[44] Venczel M, Csiki Z (2003). New frogs from the latest Cretaceous of Haţeg Basin, Romania. Acta Palaeontol. Pol..

[45] Maganuco S (2009). An exquisite specimen of *Edingerella madagascariensis* (Temnospondyli) from the Lower Triassic of NW Madagascar: cranial anatomy, phylogeny, and restorations. Museo. Civico. di Storia. Natural. di Milano..

[46] Ascarrunz E, Rage JC, Legreneur P, Laurin M (2016). *Triadobatrachus massinoti*, the earliest known lissamphibian (Vertebrata: Tetrapoda) re-examined by μCT scan, and the evolution of trunk length in batrachians. Contrib. Zool..

[47] Roček Z, Rage JC (2000). Anatomical transformations in the transition from temnospondyl to proanuran stages. Amphib. Biol..

[48] Swofford, D. L. PAUP*. Phylogenetic Analysis Using Parsimony (*and OtherMethods) (Sinauer, Sunderland, MA), Version4.0 (2002).

