# Supplementary material for: The earliest direct evidence of frogs in wet tropical forests from Cretaceous Burmese amber
Source: Sci Rep. 2018 Jun 14;8:8770. doi: 10.1038/s41598-018-26848-w (PMC6002357; doi:10.1038/s41598-018-26848-w)
Supplement: Supplementary file 1 — Supplementary Materials [file 41598_2018_26848_MOESM1_ESM.pdf]

Supplemental Materials
Xing L, Stanley EL, Bai M, Blackburn DC. The earliest direct evidence of frogs in wet tropical forests from Cretaceous Burmese amber.

Supplemental figures

Supplemental figure 1. Photographs of 3D-printed replica (FLMNH VP-312847) in (a) dorsal, (b) lateral, and (c) anterior views. Photographs of this replica were taken by Kristen Grace (Florida Museum of Natural History).

Supplemental video 1. Video showing x-ray tomograms of holotype of *Electrorana* (DIP-L-0826), beginning at the rostrum and scrolling posteriorly.

Supplemental video 2. Video showing x-ray tomograms of holotype of *Electrorana* (DIP-L-0826), beginning at the left lateralmost extent of the specimens and scrolling right.

Supplemental video 3. Video showing regions of interest of the holotype of *Electrorana* (DIP-L-0826) based on discussion in the manuscript.

Supplementary Table 1. Specimens examined

Family	Scientific Name	Catalog #	MorphoSource DOI	Extant/Extinct
Undetermined	*Electrorana limoae*	DIP-V-16119	doi:10.17602/M2/M29450	Extinct
Undetermined	*Electrorana limoae*	DIP-V-16121	doi:10.17602/M2/M29451	Extinct
Undetermined	*Electrorana limoae*	DIP-V-16127	doi:10.17602/M2/M29452	Extinct
Undetermined	*Electrorana limoae*	DIP-L-0826	doi:10.17602/M2/M29447	Extinct
Undetermined	*Callobatrachus sanyanensis*	IVPP V11525		Extinct
Undetermined	*Liaobatrachus zhaoi*	IVPP V14203		Extinct
Undetermined	*Mesophryne beipiaoensis*	IVPP V12717		Extinct
Undetermined	*Yizhoubatrachus macilentus*	IVPP V12510		Extinct
Alytidae	*Alytes obstetricans*	CAS-SUA-21691	doi:10.17602/M2/M24013	Extant
Alytidae	*Discoglossus pictus*	KU-H-144217	doi:10.17602/M2/M29730	Extant
Ascaphidae	*Ascaphus truei*	UF-H-80664	doi:10.17602/M2/M11258	Extant
Bombinatoridae	*Barbourula busuangensis*	UF-H-70546	doi:10.17602/M2/M16092	Extant
Bombinatoridae	*Bombina maxima*	UF-H-96648	doi:10.17602/M2/M23561	Extant
Heleophrynidae	*Hadromophryne natalensis*	UF-H-100828	doi:10.17602/M2/M12695	Extant
Leiopelmatidae	*Leiopelma hamiltoni*	CAS-H-53931	doi:10.17602/M2/M24351	Extant
Megophryidae	*Brachytarsophrys carinensis*	UF-H-63888	doi:10.17602/M2/M16763	Extant
Megophryidae	*Leptobrachium hasseltii*	UF-H-61841	doi:10.17602/M2/M16288	Extant
Pelobatidae	*Pelobates fuscus*	UF-H-36935	doi:10.17602/M2/M24459	Extant
Pelobatidae	*Pelobates varaldii*	CAS-H-92371	doi:10.17602/M2/M25442	Extant
Pelodytidae	*Pelodytes caucasicus*	CAS-H-94059	doi:10.17602/M2/M24582	Extant
Pipidae	*Pipa parva*	UF-H-37924	doi:10.17602/M2/M12424	Extant
Pipidae	*Xenopus clivii*	UF-H-92704	doi:10.17602/M2/M26262	Extant
Rhinophrynidae	*Rhinophrynus dorsalis*	CAS-H-71766	doi:10.17602/M2/M16302	Extant
Scaphiopodidae	*Scaphiopus holbrooki*	UF-H-9620	doi:10.17602/M2/M22642	Extant
Scaphiopodidae	*Spea multiplicata*	UF-H-100788	doi:10.17602/M2/M11082	Extant

Characters from Henrici et al. (2013)

For discussion of character states and codings, please see Henrici et al. (2013).

1. Dermal sculpture: (0) absent; (1) present.
2. Nasals, medial contact: (0) well separated; (1) in contact or very narrowly separated.
3. Frontoparietal, relationship with nasal: (0) not in contact; (1) in contact.
4. Frontoparietals, relationship to one another: (0) separate throughout length; (1) in partial contact; (2) sutured together throughout length and/or partially fused; (3) fused with no trace of suture.
5. Frontoparietal, supraorbital flange: (0) absent; (1) present.
6. Planum antorbitale, ossification: (0) completely cartilaginous or ossified/mineralized less than one-half; (1) ossified/mineralized more than one-half.
7. Perilymphatic foramina, position: (0) superior perilymphatic foramen anterior to jugular foramen, inferior perilymphatic foramen posterior to jugular foramen; (1) both superior and inferior perilymphatic foramina posterior to jugular foramen.
8. Maxilla-premaxilla relationship: (0) slight or no overlap; (1) pointed process of pars facialis of maxilla that reaches alary process of premaxilla.
9. Maxilla, preorbital process: (0) absent or weakly developed; (1) present and well developed.
10. Maxilla, palatine process of pars facialis: (0) absent; (1) present.
11. Maxilla, length: (0) does not extend beyond midlength of orbit; (1) extends beyond midlength of orbit.
12. Quadratojugal: (0) present; (1) absent.
13. Vomer: (0) present; (1) absent.
14. Vomer, contact with inner surface of pars facialis of maxilla: (0) absent; (1) present.
15. Vomer, postchoanal process: (0) short; (1) elongate and articulates with pars facialis of pars palatina of maxilla; (2) absent.
16. Palatine: (0) absent; (1) present.
17. Parasphenoid, anterior extent of cultriform process: (0) does not reach antorbital plane; (1) reaches antorbital plane but not maxillary arcade; (2) reaches maxillary arcade.
18. Parasphenoid, lateral alae: (0) present; (1) absent.
19. Parasphenoid, posteromedial process: (0) absent; (1) present.
20. Parasphenoid, posteromedial process shape: (0) posteriorly directed apex; (1) distally rounded; (2) distally rectangular; (3) distally concave.
21. Squamosal shape: (0) generally triradiate; (1) funnel shaped.
22. Squamosal, zygomatic ramus, contact with maxilla: (0) absent; (1) present.
23. Squamosal, otic ramus: (0) greatly reduced to absent; (1) present, forming distinct process.
24. Squamosal, otic plate: (0) reduced to absent; (1) well developed.
25. Crista parotica: (0) poorly developed; (1) well developed.
26. Occipital artery: (0) dorsal to skull roof; (1) in closed canal that exits laterally; (2) in closed canal that exits dorsally.

27. Pterygoid, anterior ramus length: (0) short, less than twice the length of medial ramus; (1) elongate, greater than twice the length of medial ramus.
28. Pterygoid, medial ramus contact with parasphenoid lateral ala: (0) contact; (1) no contact.
29. Pterygoid, ventral flange: (0) present; (1) absent.
30. Angulosplenial, coronoid process: (0) poorly developed; (1) present and blade-like; (2) present and knob-like (thumb-like).
31. Jaw articulation, position: (0) lateral to otic capsule; (1) posterior to otic capsule; (2) at anterior margin of otic capsule.
32. Hyoid apparatus, posteromedial process, anterior end width: (0) wider than posterior end; (1) narrower than or equal to posterior end.
33. Hyoid apparatus, parahyoid bone: (0) present; (1) absent.
34. Hyoid apparatus, parahyoid bone shape: (0) single; (1) paired.
35. Hyoid apparatus, hyale general configuration: (0) complete; (1) incomplete.
36. Atlas, cotyles configuration: (0) closely juxtaposed or forming single articular surface; (1) widely separated.
37. Vertebral centra, formation: (0) perichordal; (1) epichordal.
38. Vertebral centra, articular facet of last presacral vertebra: (0) opisthocoelous; (1) procoelous; (2) amphicoelous.
39. Presacral vertebrae VI and VII, posterior margin of the neural arch: (0) slightly concave to straight or with a minute spine; (1) projecting in a well-developed spine; (2) deeply notched.
40. Presacral vertebrae I and II, relationship of neural arches: (0) not fused, weak or no imbrication; (1) not fused, broad imbrication medially only; (2) not fused, imbrication involving all of neural lamina; (3) synostotically fused to form long, combined element.
41. Presacral vertebrae IV and V, transverse process relative length: (0) transverse process length of presacral IV less than that of presacral V; (1) transverse process length of presacral IV is greater than that of presacral V; (2) transverse process length of presacral IV is subequal to that of presacral V.
42. Presacral vertebra VI, orientation of the posterior margin of the transverse processes relative to the axial axis: (0) nearly perpendicu- lar; (1) moderately anteriorly; (2) strongly anteriorly; (3) moderately posteriorly.
43. Ribs: (0) free ribs present in adults; (1) free ribs absent in adults.
44. Sacrum and urostyle articulation: (0) monocondylar; (1) bicondylar; (2) fused; (3) strap-like.
45. Sacrum, diapophyses, distal cross section: (0) flattened; (1) subcircular.
46. Sacrum, diapophyses expansion: (0) broadly expanded, length greater than or equal to combined process width; (1) expanded, length greater than half process width but less than combined process width; (2) not expanded, length less than half process width.
47. Sacrum, diapophyses lateral margins: (0) straight; (1) convex.
48. Clavicle, lateral end relationship to scapula: (0) contacts medial edge of pars acromialis; (1) overlaps anterior edge of pars acromialis; (2) fused to scapula.
49. Scapula, proportions: (0) dorsoventral length of glenoid area one-third or less dorsoventral length of scapula; (1) dorsoventral length of glenoid area greater

than one-third dorsoventral length of scapula.

50. Scapula-clavicle, proportions: (0) scapula shorter than clavicle; (1) scapula longer than clavicle; scapula of subequal length to clavicle (2).
51. Scapula, anterior tubercle: (0) absent; (1) present.
52. Scapula, anterior lamina: (0) present; (1) absent.
53. Cleithrum, distal margin: (0) bifurcate; (1) not bifurcate.
54. Coracoid, sternal expansion relative to coracoid length: (0) sternal expansion less than half the length of coracoid; (1) sternal expansion greater than half the length of coracoid.
55. Omosternum, presence: (0) present; (1) absent.
56. Sternum, condition: (0) cartilaginous; (1) ossified.
57. Sternum, shape: (0) linear; (1) triradiate.
58. Postaxial carpals (ulnare and distals 3, 4, and 5), configuration: (0) all free; (1) ulnare and 3 free, 4 and 5 fused; (2) ulnare free, 3, 4, and 5 fused; (3) ulnare fused to 5, 4 free
59. Carpal torsion: (0) absent; (1) present.
60. Iliac shaft, spiral groove: (0) absent; (1) present.
61. Iliac shaft, dorsal crest: (0) absent; (1) present as low ridge; (2) present as well-developed flange.
62. Dorsal crest, position on iliac shaft: (0) restricted to distal half of shaft; (1) restricted to proximal portion of shaft; (2) extends along nearly entire length of shaft.
63. Ischium, shape in lateral aspect: (0) long with subrectangular outline; (1) short with convex distal margin and semi-circular outline.
64. Tibiofibula, length: (0) shorter than femur; (1) of subequal length with femur; (2) longer than femur.
65. Tibiale-fibulare relationship: (0) not fused or fused only at proximal and distal ends; (1) fused throughout length to form single bone.
66. Prehallux, distal bone: (0) not modified into spade; (1) modified into spade.

Characters from Báez (2013)
For discussion of character states and codings, please see Báez (2013).

1. Preorbital region, relative length: (0) one-third, or more, of the skull length; (1) one-quarter, or less, of the skull length.
2. Nasals, relationship to one another: (0) broadly separated; (1) narrowly separated or sutured.
3. Frontoparietals, relationship with nasals: (0) not in contact; (1) in contact.
4. Frontoparietals, relationship to one another: (0) separate throughout most of their lengths; (1) posteromedial margins close or in contact, diverging only anteriorly; (2) sutured throughout length; (3) completely fused or azygous.
5. Frontoparietals, supraorbital flange: (0) absent; (1) present.
6. Pineal foramen: (0) absent; (1) present.
7. Sphenethmoid, configuration: (0) right and left elements dorsally and ventrally separated; (1) dorsally separated; (2) girdle-bone.
8. Sphenethmoid, dorsal exposure: (0) present; (1) absent.
9. Optic foramina, margins: (0) bound in cartilage or cartilage and bone; (1) bound completely in bone.
10. Postnasal wall, ossification: (0) completely cartilaginous or ossified/mineralized less than one-half; (1) ossified/mineralized more than one-half.
11. Perilymphatic foramina, position respect to jugular foramen (0) inferior and superior anterior; (1) inferior posterior, superior anterior; (2) both posterior
12. Prootic, Eustachian canal: (0) absent; (1) present.
13. Maxilla, preorbital process: (0) absent or weakly developed; (1) well developed.
14. Maxilla, pars facialis, palatine process: (0) absent; (1) present.
15. Quadratojugal: (0) present; (1) absent.
16. Vomer: (0) present; (1) absent.
17. Vomer, plate-like anterior portion: (0) absent or poorly developed; (1) well-developed.
18. Vomer, postchoanal process: (0) absent; (1) present.
19. Palatine: (0) absent; (1) present.
20. Parasphenoid, subotic alae: (0) present; (1) absent.
21. Parasphenoid, cultriform process, anterior extent: (0) not reaching maxillary arch; (1) reaching maxillary arch.
22. Squamosal, zygomatic ramus: (0) reduced or absent; (1) moderately to well developed, free-ending; (2) well developed, articulating with maxilla; (3) well developed, articulating with pterygoid.
23. Squamosal, otic ramus: (0) poorly to moderately developed, medial process poorly differentiated or absent; (1) present, with distinct medial process that rests on the crista parotica; (2) present, with extensive medial process that rests on the otoccipital.
24. Crista parotica, condition: (0) mostly cartilaginous; (1) mineralized.
25. Occipital artery, location: (0) dorsal to skull roof; (1) housed in a closed canal.
26. Pterygoid, medial ramus, contact with parasphenoid: (0) absent; (1) present.
27. Pterygoid, anterior ramus, length: (0) short to moderate, anterior end well posterior to the antorbital plane; (1) long, reaching or nearly, the antorbital plane.

28. Angulosplenial, coronoid process: (0) low, poorly developed; (1) well developed, but short; (2) long, blade-like.
29. Mentomeckelian bone: (0) present; (1) absent.
30. Jaw articulation, position: (0) lateral to the otic capsule; (1) at the anterolateral corner of the otic capsule.
31. Hyoid apparatus, posteromedial process, anterior end: (0) wider than posterior end; (1) equally developed or narrower than posterior end.
32. Hyoid apparatus, parahyoid bone: (0) present; (1) absent.
33. Hyoid apparatus, hyale general configuration: (0) complete; (1) incomplete.
34. Presacral vertebrae, number: (0) nine; (1) eight; (2) seven (eighth vertebra incorporated into the sacrum).
35. Atlas, cotyle configuration: (0) closely juxtaposed (including a single articulation facet); (1) well separated (type I of Lynch, 1971).
36. Vertebral centra, formation pattern: (0) perichordal; (1) epichordal.
37. Last presacral vertebra, configuration of centrum according to articulation facets: (0) notochordal; (1) opisthocoelous; (2) procoelous; (3) amphicoelous.
38. Posterior presacral vertebrae (except last presacral), posteromedial margin of neural arch: (0) slightly concave to straight or with a minute neural spine; (1) projecting in a well-developed neural spine.
39. Vertebra VI, transverse processes, proximal-distal length in comparison to sacral length: (0) shorter; (1) nearly equal.
40. Presacral vertebrae I and II: (0) not fused, weak or no imbrication; (1) not fused, broad imbrication medially only; (2) not fused, broad imbrication involving all the neural laminae; (3) synostotically fused; (4) synchondrotically fused.
41. Vertebra VI, orientation of posterior margin of transverse processes with respect to axial axis: (0) nearly perpendicular; (1) moderately forward; (2) markedly forward: (3) posterior.
42. Ribs on anterior presacrals: (0) free bony ribs present in larvae and adults; (1) free bony ribs present in larvae and ankylosed to transverse processes in adults; (2) free bony ribs absent in larvae and adults.
43. Sacrum and urostyle, articulation: (0) non-synovial; (1) synovial, monocondylar; (2) synovial, bicondylar; (3) synchondrotically or synostotically fused.
44. Sacral diapophyses, distal cross-section: (0) flattened; (1) nearly round
45. Sacral diapophyses, distal expansion as ratio of distal length and mediolateral width: (0) widely expanded (ratio ! 1.5); (1) moderately expanded (1.5 > ratio > 0.75); (2) weakly expanded or unexpanded (ratio < 075).
46. Sacral diapophyses, distal margin in dorsal view: (0) laterally convex; (1) straight
47. Sacral diapophyses, anterior margin orientation in dorsal view: (0) nearly horizontal; (1) anterior; (2) posterior.
48. Urostyle, transverse processes: (0) present; (1) absent.
49. Urostyle, dorsal ridge: (0) absent, inconspicuous or low; (1) present, moderately to well developed.
50. Clavicle, relation to scapula: (0) lateral end contacts medial edge of pars acromialis; (1) lateral end overlaps anterior edge of pars acromialis; (2) lateral end is fused to scapula.

51. Scapula, proportions: (0) glenoid area one-third, or less, total length; (1) glenoid area more than one-third total length.
52. Scapula, relative length: (0) shorter than clavicle; (1) longer than clavicle; (2) nearly as long as clavicle.
53. Scapula, anterior lamina: (0) present; (1) absent.
54. Cleithrum, distal end: (0) not forked; (1) forked.
55. Coracoid, sternal expansion relative to coracoid length: (0) sternal expansion less than half the length of the coracoid; (1) sternal expansion nearly half the length of the coracoid; (2) sternal expansion nearly the length of the coracoid.
56. Omosternum: (0) present; (1) absent.
57. Omosternum, condition: (0) cartilaginous; (1) ossified, not forked; (2) ossified, forked.
58. Sternum: (0) present; (1) absent.
59. Sternum, condition: (0) cartilaginous; (1) ossified.
60. Humerus, shaft: (0) columnar; (1) ventrally curved.
61. Post axial carpals (ulnare, distal carpals 3, 4, and 5): (0) all discrete; (1) distal carpals 4 and 5 fused; (2) distal carpals 3, 4, and 5 fused.
62. Carpal torsion: (0) absent; (1) present.
63. Metacarpals, length: (0) relatively short, not longer than 60 % of radioulna length; (1) long, longer than 70 % of radioulna length.
64. Ilium, spiral groove on shaft: (0) absent; (1) present.
65. Ilium, dorsal crest on shaft: (0) absent; (1) present as a low ridge; (2) well developed as a flange (wider than one-half of the shaft width).
66. Interiliac scar: (0) absent to narrow; (1) ample, but restricted to ventral part of ilia; (2) ample both ventrally and dorsally.
67. Angle between the margin of the ventral acetabular expansion and the ventral margin of the iliac shaft in acetabular view: (0) acute; (1) nearly right; (2) obtuse.
68. Ischium, shape in lateral view: (0) long, with a nearly rectangular outline; (1) short, with semicircular outline.
69. Pubis, condition: (0) cartilaginous; (1) partially or completely ossified.
70. Tibiofibula, length in relation to femur length: (0) shorter; (1) subequal; (2) longer.
71. Prehallux, distal bone: (0) not modified; (1) modified into a spade.
72. Distal tarsals 2 and 3: (0) separate; (1) fused.

Characters from Gao & Chen (2017)
For discussion of character states and codings, please see Gao & Chen (2013).

1. Shape of the skull in dorsal aspect: skull apparently wider than long (0); or roughly as long as wide, or longer (1). Remarks: Modified from Cannatella (1985) (character 1) and Gao and Wang (2001) (character 1).
2. Sculpture on dermal skull roof: absent or only weakly present (0); or present, with a pitted pattern (1); or present, with a grooved pattern (2). Remarks: Modified from Cannatella (1985) (character 3) and Gao and Wang (2001) (character 2).
3. Medial contact of nasals: contact present (0); or contact absent (1); or nasal fused medially (2). Remarks: Modified from Gao and Wang (2001) (character 3).
4. Anterolateral margin of nasal: nasal with a concave anterolateral margin for embracing the narial opening (0); or nasal more circular, with essentially a straight anterolateral margin, not embracing the narial opening (1). Remarks: Modified from Cannatella (1985) (character 5).
5. Distinct rostral process of nasal: present (0); or absent (1). Remarks: Modified from Gao and Wang (2001) (character 5).
6. Extent of posterior divergence of nasals: divergence minimal, involving less than half the length of nasals (0); or divergence extensive, involving over half the length of nasals (1). Remarks: Modified from Maglia (1998) (character 9).
7. Ossification of septum nasi: septum nasi cartilaginous (0); or septum nasi bony posteriorly, extending about one-half the length of the nasals (1); or septum nasi bony along most of the length of the nasals (2). Remarks: Modified from Cannatella (1985) (character 15).
8. Fusion of frontal and parietal: frontal and parietal remain separate (0); or fused to form frontoparietal (1). Remarks: Modified from Gao and Wang (2001) (character 6).
9. Fusion of two frontoparietal medially in adults: frontoparietal paired without fusion (0); or azygous frontoparieal present due to fusion (1). Remarks: Modified from Cannatella (1985) (character 21) and Gao and Wang (2001) (character 7).
10. Dorsal exposure of frontoparietal fontanelle: fontanelle not exposed (0); exposed 50% of its length or less (1); or exposed more than 50% of its length (2). Remarks: Modified from Cannatella (1985) (character 22) and Gao and Wang (2001) (character 8).
11. Posterolateral process (margo prootica) of frontoparietal: well developed and wing-like (0); or poorly developed (1); or completely absent (2). Remarks: Modified from Gao and Wang (2001) (character 9).
12. Supraorbital flange of frontoparietal: absent (0); or present (1). Remarks: Modified from Cannatella (1985) (character 25) and Gao and Wang (2001) (character 10).
13. Contact between frontoparietal and nasal: contact present (0); contact absent (1). Remarks: Modified from Trueb (1993) (character 4).
14. Formation of prootic-occipital region: by prootic-exoccipital-opisthotic complex (0); by prootic-exoccipital without fusion (1); or by fused prootic-exoccipital (2). Remarks: Modified from Gao and Wang (2001) (character 11).
15. Perilymphatic foraman: double foramina open on medial capsular wall (0); or

double foramina present on posterior wall of otic capsule (1); only superior perelymphatic foramen present (2); or only inferior foramen present (3). Remarks: Modified from Gao and Wang (2001) (character 12).

16. Width of alary process of premaxilla: thin, with one fouth or less width of premaxilla (0); one third or greater width of premaxilla (1). Remarks: Modified from Maglia (1998) (character 12).
17. Palatine process of premaxilla: absent or barely present (0); or well developed (1). Remarks: Modified from Cannatella (1985) (character 52) and Gao and Wang (2001) (character 13).
18. Premaxilla-maxilla articulation: posterior process of premaxilla absent (0); or present (1). Remarks: Modified from Gao and Wang (2001) (character 14).
19. Posterior extent of maxilla: maxilla long, extending posteriorly for most of the length of the orbit (0); or maxilla relatively short, not extending posteriorly beyond half the length of orbit (1). Remarks: Modified from Maglia (1998) (character 18).
20. Premaxilla-nasal articulation: articulation present (0); or articulation lost with separation of the two elements (1). Remarks: Modified from Gao and Wang (2001) (character 15).
21. Prefrontal and anterior margin of the orbit: prefrontal present, maxilla and nasal excluded from the anterior margin of the orbit (0); prefrontal lost with maxilla and nasal forming the anterior margin of the orbit (1); nasal forming most of the anterior margin of the orbit (2); or anterior ramus of pterygoid excluding maxilla from the anterior margin of the orbit (3). Remarks: Modified from Gao and Wang (2001) (character 16).
22. Quadratojugal: present (0); or absent (1). Remarks: Modified from Cannatella (1985) (character 62) and Gao and Wang (2001) (character 17).
23. Shape of squamosal: as a simple horizontal bar (0); or triradiate and T-shaped (1); or nontriradiate with loss of zygomatic ramus (2); or funnel shaped in fusion with tympanic annulus (3). Remarks: Modified from Cannatella (1985) (character 40, 41, 42) and Gao and Wang (2001) (character 18).
24. Squamosal-maxilla contact: absent (0); or contact present (1). Remarks: Modified from Cannatella (1985) (character 43) and Gao and Wang (2001) (character 19).
25. Expansion of otic ramus of squamosal in lateral view: not expanded (0); or otic ramus expanded and deep (1). Remarks: Modified from Gao and Wang (2001) (character 20).
26. Medial articulation of squamosal: squamosal medially in contact with dermal skull table (0); or squamosal not in contact with dermal skull table, but articulating with the crista prootica (1). Remarks: Modified from Gao and Wang (2001) (character 21).
27. Sphenethmoid: bilaterally paired (0); or single (1). Remarks: Modified from Cannatella (1985) (character 17) and Gao and Wang (2001) (character 22).
28. Vomers: present, paired (0); or absent (1), or present, azygous (2). Remarks: Modified from Cannatella (1985) (character 8, 9).
29. Position of anterior process of vomer: anterior process of vomer lying immediately behind premaxilla (0); or lying near premaxilla-maxilla articulation (1). Remarks: Modified from Gao and Wang (2001) (character 23).

30. Postchoanal process of vomer: absent (0); or present, forming wide angle (about 90-110°) with anterior portion of vomer (1); or present, forming narrow angle (about 45°) with anterior portion of vomer (2). Remarks: Modified from Gao and Wang (2001) (character 24).
31. Elongation of the postchoanal process of vomer: not elongate (0); or elongate (1). Remarks: Modified from Cannatella (1985) (character 11).
32. Palatine: present as discrete element (0); or absent (1). Remarks: Modified from Cannatella (1985) (character 12) and Gao and Wang (2001) (character 25).
33. Anterior terminus of cultriform process of parasphenoid: extending anteriorly to the level of the vomers (0); or not reaching the level of the vomers (1). Remarks: Modified from Gao and Wang (2001) (character 26).
34. Posterolateral alae of parasphenoid: anteroposterior width of alae equal or greater than one-third distance between lateral ends (0); or width narrower than one-third distance between lateral ends (1); or alae absent (2). Remarks: Modified from Gao and Wang (2001) (character 27).
35. Posterolateral notch of parasphenoid alae: present (0); or absent (1). Remarks: Modified from Gao and Wang (2001) (character 28).
36. Posteromedial process of parasphenoid: absent, leaving the posterior border of parasphenoid straight or concave (0); or present (1). Remarks: Modified from Gao and Wang (2001) (character 29).
37. Relationships of parasphenoid and sphenethmoid: two elements separate (0); or at least partially fused (1). Remarks: Modified from Cannatella (1985) (character 33).
38. Medial ramus of pterygoid: not contacting parasphenoid (0); or contacting parasphenoid (1); or medial ramus absent (2). Remarks: Modified from Gao and Wang (2001) (character 30).
39. Ventral flange of the anterior ramus of the pterygoid: absent (0); or present as a ventrally directed flange (1). Remarks: Modified from Cannatella (1985) (character 36).
40. Parahyoid bone: present and single (0); or present and paired (1); or absent (2). Remarks: Modified from Cannatella (1985) (character 170, 171) and Gao and Wang (2001) (character 31).
41. Columella: well-ossified columella present (0); or absent (1); or present, but reduced in size (2). Remarks: Modified from Cannatella (1985) (character 45, 46) and Gao and Wang (2001) (character 32).
42. Mentomeckelian bone ossification: present (0); or absent (1). Remarks: Modified from Cannatella (1985) (character 66) and Gao and Wang (2001) (character 33).
43. Upper marginal teeth: present (0); or absent (1).
44. Lower marginal teeth: present (0); or absent (1).
45. Occipital foraman: pathway for occipital vessels open on frontoparietal (0); or pathway for occipital vessels roofed by bone (1). Remarks: Modified from Maglia (1998) (character 5).
46. Number of presacral vertebrae: 14 or more (0); ten presacral vertebrae (1); normally nine presacral vertebrae (2); normally eight or few (3). Remarks: Modified from Gao and Wang (2001) (character 35).
47. Fusion of presacrals I and II: fusion absent (0); or fusion present (1). Remarks:

Modified from Cannatella (1985) (character 76) and Gao and Wang (2001) (character 36).

48. Centrum of presacral vertebrae: vertebral centra amphicoelous or notochordal (0); or opisthocoelous (1); or procoelous (2). Remarks: Modified from Gao and Wang (2001) (character 37).
49. Neural arch of presacral vertebrae: completely or weakly imbricated roofing of spinal canal (0); or not imbricated with spinal canal partially exposed (1). Remarks: Modified from Gao and Wang (2001) (character 38).
50. Morphology of atlantal cotyles: cotyles mostly ventral and narrowly separated by notochordal fossa (0); cup-like cotyles displaced laterally and widely separated from one another (1); cotyles confluent as a single articular surface (2). Remarks: Modified from Gao and Wang (2001) (character 39).
51. Free ribs on presacral vertebrae: free ribs present on all presacral vertebrae (0); ribs present on presacral II-V or II-VI (1); or ribs restricted to presacrals II-IV (2); or present on presacrals II-IV till subadult stage (3); free ribs absent in both subadults and adults (4). Remarks: Modified from Cannatella (1985) (character 80) and Gao and Wang (2001) (character 40).
52. Length of transverse process: transverse process of verbetra II longest, or of equal length of III and IV (0); or transverse process of vertebra III longest (1); or that of IV longest (2). Remarks: Modified from Maglia (1998) (character 40).
53. Transverse process of posterior presacral vertebrae: more laterally than anterolaterally oriented (0); or essentially anterolaterally oriented (1). Remarks: Modified from Gao and Wang (2001) (character 41).
54. Fusion of sacral ribs: remain free from sacral vertebra (0); or fused to transverse process of sacrum (1). Remarks: Modified from Gao and Wang (2001) (character 42).
55. Dilation of sacral diapophysis: slender with little or no dilation (0); or moderately dilated and hatchet-shaped, with a convex lateral edge (1); or widely expanded as butterfly wing-shaped, with more or less a straight lateral edge (2). Remarks: Modified from Cannatella (1985) (character 103) and Gao and Wang (2001) (character 43).
56. Postsacral vertebrae: caudal vertebrae remain unfused (0); or urostyle present in association with discrete caudal between sacrum and urostyle (1); or all postsacral vertebrae uniformly modified into single urostyle (2). Remarks: Modified from Gao and Wang (2001) (character 44).
57. Relative length of urostyle: shorter than combined length of presacral vertebrae (0); or as long or longer than combined length of presacral vertebrate (1). Remarks: Modified from Maglia (1998) (character 46).
58. Sacral-urostyle articulation: cartilaginous joint (0); bicondylar (1); monocondylar (2); or simply fused (3). Remarks: Modified from Cannatella (1985) (character 83) and Gao and Wang (2001) (character 45).
59. Transverse process on postsacral complex: present (0); or fused to a bony web of sacral diapophysis (1); or absent (2). Remarks: Modified from Gao and Wang (2001) (character 46).
60. Dorsal Crest on urostyle: absent (0); present, extending to half-length of urostyle (1); present, extending to almost the full length of urostyle (2).

61. Type of pectoral girdle: arciferal, with the epicoracoid cartilages overlapping one another and the sternum attached to the pectoral arch (0); or firmisternal, with the epicoracoid fused to some degree along the midline (1). Remarks: Modified from Cannatella (1985) (character 88).
62. Presence of prezonal element: absent (0); or present as a cartilaginous plate (1); or present as a bony style (2). Remarks: Modified from Cannatella (1985) (character 85).
63. Posterior ends or epicoracoid cartilages: not expanded (0); or expanded to the level of lateral edge of the sternum (1). Remarks: Modified from Cannatella (1985) (character 87).
64. Length of scapula: at least half-length of humerus (0); or less than half-length of humerus (1). Remarks: Modified from Gao and Wang (2001) (character 47).
65. Overall shape of scapula: short and stocky (0); or relatively long, about two to three times as long as it is wide (1). Remarks: Modified from Cannatella (1985) (character 100).
66. Leading edge of scapula: leading edge concave (0); or straight (1). Remarks: Modified from Gao and Wang (2001) (character 48).
67. Anterior overlap of clavicle on scapula: overlap absent (0); or overlap present (1); or clavicle and pars acromialis of scapula fused (2). Remarks: Modified from Gao and Wang (2001) (character 49).
68. Curvature of long axis of clavicle: straight or only slightly bowed (0); or strongly bowed (1).
69. Sternal end of clavicle: narrower than the body of clavicle (0); or sternal end expanded and broader than the body of clavicle (1). Remarks: Modified from Cannatella (1985) (character 95).
70. Medial end of coracoid: medial end little or slightly expanded, narrower than distal end (0); or medial end of coracoid greatly expanded, wider than the distal end, and usually have an arched edge (1). Remarks: Modified from Gao and Wang (2001) (character 50).
71. Relative lengths of clavicle/coracoid: clavicle approximately equal in length to coracoid (0); or clavicle much longer than coracoid (1). Remarks: New character. Polarity is tentative due to unknown condition in *Triadobatrachus*.
72. Cleithrum: present and unforked (0); present and forked (1); or cleithrum fused to suprascapula (2). Remarks: Modified from Gao and Wang (2001) (character 51).
73. Bony sternum stylus: absent (0); or present (1). Remarks: Modified from Cannatella (1985) (character 91).
74. Condition of sternal plate: sternum absent (0); sternum forming elongate rod (1); sternum forming semicircle with concave anterior margin (2); or sternum forming thin, sickle shape (3). Remarks: Modified from Maglia (1998) (character 49).
75. Humeral condyle: single condyle with small diameter less than 60% of distal width (0); or single condyle enlarged with diameter greater than 60% of distal width (1). Remarks: Modified from Gao and Wang (2001) (character 52).
76. Ossification of humeral condyle: condyle unossified (0); or condyle ossified (1). Remarks: Modified from Gao and Wang (2001) (character 53).
77. Epipodial elements: remaining as separate elements (0); or fused to form single element (1). Remarks: Modified from Gao and Wang (2001) (character 54).

78. Free intermedium in carpus: present (0); or absent, by fusion with ulnare (1). Remarks: Modified from Gao and Wang (2001) (character 55).
79. Fusion of distal carpal III and IV with postaxial centrale: absent, distal carpals III and IV free (0); or distal carpal IV fused with to postaxial centrale (1); or distal carpal III and IV both fused to postaxial centrale (2). Remarks: Modified from Cannatella (1985) (character 135).
80. Length and orientation of ilium: short ilium essentially dorsally directed (0); or elongate shaft of ilium anteriorly directed (1). Remarks: Modified from Gao and Wang (2001) (character 56).
81. Dorsal acetabular expansion of ilium: not extending to dorsal limit of ischium (0); or extending to dorsal limit of ischium (1). Remarks: Modified from Gao and Wang (2001) (character 57).
82. Dorsal tubercle of ilium: strongly developed as a distinct tubercle (0); or weakly developed as a low process (1); or essentially absent (2). Remarks: Modified from Cannatella (1985) (character 109) and Gao and Wang (2001) (character 58).
83. Dorsal crest on body of ilium: absent (0); or present, dorsoventrally directed (1); or present, laterally directed (2). Remarks: Modified from Cannatella (1985) (character 104).
84. Ossification of pubis: pubis remains cartilaginous (0); pubis ossified (1). Remarks: Modified from Cannatella (1985) (character 111).
85. Hind limb proportions: similar or only slightly longer than front limb (0); or proportionally longer (1). Remarks: Modified from Gao and Wang (2001) (character 59).
86. Epipubis: absent (0); or present as a large plate (1); or present as a narrow stripe (2). Remarks: Modified from Cannatella (1985) (character 112).
87. Condition of ventral crest of femur (*crista femoris*): absent or poorly developed (0); or present (1). Remarks: Modified from Cannatella (1985) (character 115).
88. Fusion of proximal tarsals: fusion absent (0); or fused at proximal and distal ends (1); or completely fused to form a single bone (2). Remarks: Modified from Gao and Wang (2001) (character 60).
89. Number of tarsalia: three or more free elements (0); or only two elements present (1). Remarks: Modified from Gao and Wang (2001) (character 61).
90. Prehallux: absent (0); or present as small hind foot element (1); or modified as bony spade (2). Remarks: Modified from Cannatella (1985) (character 151) and Gao and Wang (2001) (character 62).
91. Shape of prehallux: sub-oval (0); elongate, scaphoid-shaped (1); or cuneiform-shaped (2). Remarks: Modified from Cannatella (1985) (character 152).
92. Consolidation of Cranial Nerve V and VII: three separate foramina occur (0); or trigeminal and facial foramina separated by prefacial commissure (1); or commissure absent, nerva exit via single prootic foramen (2). Remarks: Modified from Gao and Wang (2001) (character 63).
93. Posture of manus: medial inturning of first finger absent (0); or inturning of the first finger present (1). Remarks: Modified from Cannatella (1985) (character 133) and Gao and Wang (2001) (character 65).
94. Depressor mandibulae: consisting of one head or two slightly divided parts witho origin from the dorsal fascia (0); consisting of two discrete bellies that are at least

partially separated by the insertion of the cucullaris (1). Remarks: Modified from Cannatella (1985) (character 69).

95. Condition of the *depressor mandibulae muscle*: it originates at least in part from the otic region, either from fascia or bone (0); or it originates only from the fascia over the suprascapula (1). Remarks: Modified from Cannatella (1985) (character 70).

96. Separation of *m. semitendinosus* from *m. sartorius*: *m. sartorius* not completely distinct, at least fused to *m. semitendinosus* to some degree (0); *m. sartorius* completely dinstinct from *m. semitendinosus* (1) Remarks: Modified from Cannatella (1985) (character 132).

97. Presence of accessory head of *m. adductor magnus*: abent (0); present (1). Remarks: Modified from Cannatella (1985) (character 122).

Supplementary References from Gao & Chen (2017) related to characters:

Maglia, A. M. 1998. Phylogenetic relationships of extant pelobatoid frogs (Anura: Pelobatoidea): evidence from adult morphology. *Scientific Papers of the University of Kansas Natural History Museum* 10, 1–19.

Trueb, L. 1993. Pp. 255–343 in *The Skull, Volume 2: Patterns of Structural and Systematic Diversity* (eds J. Hanken & B. K. Hall), The University of Chicago Press.

Matrix in Nexus format based on Henrici et al. (2013)

```
#NEXUS
BEGIN DATA;
DIMENSIONS NTAX=27 NCHAR=66;
FORMAT DATATYPE = STANDARD MISSING = ? SYMBOLS = "0 1 2 3";
MATRIX
Aerugoamnis_paulus
010?00?0??100?0?????00100?1?0?01???0??0?11100?1010?10?????10?0100
Alytes_cisternasii
01010000000100000100?00001011110?0100100120010111100000100000?1100
Artroleptis_adolfifriederici
000000?00010002110110 01?0?111?011?01010023 1112?001?110001210221200
Bombina_bombina
0100000000100000000100000000110?010000100?010000111000001000010?1100
Bombina_variegata
0100000000100000000120000000 11?10100001001010000111000001000010?1000
Discoglossus_pictus   01{1 2}1000001100000{1
2}00?01101010010001001011000101111000000000000221200
Elkobatrachus_brocki
0??10???01??????013?????2????????????011110011??01???11??10?1001
Eopelobates_bayeri
110211??0110?????01301111??00????????1?1121200001100?1?11??????200
Hadromophryne_natalensis
000000?010100001100?0000001111001?00010220110110 10?00000101?0??200
Hyla_versicolor   00000010{1
2}0100001001000000101112011?01?10020110210010 1000?1210100000
Leptobrachium_hasselti
000200000011000000001200011200101 11?100121111001000101010 11?110?0100
Rana_catesbeiana   0112001010100001101000111001111???000211231112{1
2}01101110??210220100
Macropelobates_osborni
1??21?0?????????????011?11?????????0?11112?000001100?1??????230001
Miopelodytes_gilmorei
1??00???0?10?????????1100???????????????121?011??0???????????0?110
Pelobates_cultripes
1113110001100100101101111100000001?10111212100000110001011 0110?1001
Pelodytes_caucasicus
000200?001?00000?0110011000?1????1?0?1001110000?1??1?0?111?10?0210
Pelodytes_ibericus   0000000001100000101100100011110101111100121{1 2 3}000{1
2}1010000111110?0210
Pipa_carvalhoi
001310?100011??021?010001?0?01211???1023??120?010000001013?02??100
Platyplectrum_ornatum
0000001010100001101000101200110 11?000103211102100 1?1100011101 30101
```

Pristimantis_wnigrum
010200101?10000110100010100l??0?1?011100231112?202?100001210221200
Rhinophrynus_dorsalis
01131100000000011??000010??102000?0?2121310021110011l1??0?00?1001
Scaphiopus_holbrooki
1112110001110110101101111100000l1?101111101201100101000010110?1001
Scutiger_mammatus
00020000010100000012000112001011?1?100121111001101111000110110?0100
Spea_bombifrons   00010100010101101011000l120{1
2}10011?100111111201101101000010110?1001
Xenopus_tropicalis
001300?100?11??021??10001?0?01211???1003??120?021???001010?010?200
Tephrodytes_brassicarvalis   11110???0110?????0??0101121????????0?1?1121{1 2
3}001?1011??????10??21?
Electrorana_limoae
000000?00?10100021????????100?1?11?1??????0????1?0???000????????1?
;

END;

Matrix in Nexus format based on Báez (2013)

```
#NEXUS
BEGIN DATA;
DIMENSIONS NTAX=27 NCHAR=72;
FORMAT DATATYPE = STANDARD MISSING = ? SYMBOLS = "0 1 2 3 4";
MATRIX
Ascaphus_truei  00{0 1}0000000 0000100000 0100000000 0000000000 300020{0
2}001 10?111?001 000000?112 00
Alytes_cisternasii  0101002000 1000001100 0001000100 ?001011001 0020101001
1200000001 0000002101 00
Barbourula_busuangensis  11120021?0 ?010001100 01010?0?02 00010?1002
1010011001 1000000000 100001200? 00
Bombina_orientalis  0101002000 1000001100 0100001100 1001011003 1010001001
1000000?00 0001012101 0?
Bombina_variegata_pachypus  0101002000 1000001100 0100001100 1001011001
1010001001 1000000?00 000?012100 00
Callobatrachus_sanyanensis  01??0?2000 ????0011?? ?10?0?1?00 ???0?11?01
102???10?1 ??01?????1 0?0?1?1??1 0?
Cordicephalus_gracilis  11130??111 ?11?101001 11?0000010 0??1?11004 0130101101
100?0????0 0?1001??10 0?
Discoglossus_pictus  0111002000 1001001100 0211000100 0001011101 1020100001
1000100000 0000201102 00
Discoglossus_sardus  0101002000 ?001001100 0211000100 ??01011102 0020100001
120010000? 000020210? 00
Eodiscoglossus_santonjae  010100?0?0 ?0??00???? ?1?1??0??0 ???1???00? 00??2?2??1
???1?????? 000??????1 0?
Gracilibatrachus_avallei  11130121?0 ?10010??01 ?100?00000 ???100?00? 01202?21?1
10001????0 0?1?1????0 0?
Hadromophryne_natalensis  0000002000 ?010000110 0110000100 0101002002
3220200100 1000000001 010000??02 01
Iberobatrachus_angelae  010?0?2000 ?01100110? ?2?1??0000 ???10???0? 00?01?20??
1?000????0 ?00?0????2 0?
Rana_catesbeiana  0112002000 2010001110 0011010102 ?101003111 32212?2110
1111201011 2100201001 01
Neusibatrachus_wilferti  11130??100 ??0?00??01 ?1?0?00000 ?0?00?2004 01202?2??1
100?0????0 ??1?1???01 0?
Palaeobatrachus_grandipes  11130?2100 1100101101 1100001001 ?0?2012004
012010?101 10001????0 ??10112?10 0?
Pelobates_cultripes  0113102001 1001001100 0221110000 0111012101 2210011100
0100100011 0101002100 10
Phrynobatrachus_acutirostris  001200?000 ?010000110 0110000100 11011?3010
32212?2110 1101201011 210020??02 01
Platyplectrum_ornatum  0000002000 201000?110 0111101100 1101002103 12201?2110
0111000000 1100101101 11
```

Pristimantis_wnigrum 0102002000 2010001110 0111011?00 1101112010 32212?0112
021000000? 210020?112 01
Rhadinosteus_parvus ???3?0???? ?00??????1 ?????????? ???1003??? ??2????1?1
1010?????? ??0?002??1 ??
Rhinophrynus_dorsalis 0113102101 1000001101 0001000001 0011003102 0220100111
120111?1?1 0100002100 11
Xenopus_tropicalis 1113011110 ?10011??01 1311001210 1101111003 2130011102
10?001?000 0110122012 01
Spea_multiplicata 0101002001 1001101100 01111{0 1}0000 111100{0 2}001
2230101100 0110000001 0101002100 11
Thoraciliacus_rostriceps 1113?1?110 ?01?1?0?01 11?0000010 ???1001004 0110101001
12??0????0 0?1?01??10 00
Wealdenbatrachus_jucarense 0?0100???? ????0????0 011??10??0 ???????11?
302?2??2010 0?1?0????1 ??0020?1?2 ??
Electrorana_limoae 00000?10?0 ?00?11?100 0????11??0 01??1????? ?0????????
00??01?1?0 ??0??????? 0?
;
END;

Matrix in Nexus format based Gao & Chen (2017)

#NEXUS
BEGIN DATA;
DIMENSIONS NTAX=52 NCHAR=97;
FORMAT DATATYPE = STANDARD GAP = - MISSING = ? SYMBOLS = "0 1 2 3 4";
MATRIX
Triadobatrachus_massinoti
000??0?1000000????0?0?0000?????0?00000000?0??0?0000?00000000???000?00??0??0
00001000?0?000????????
Czatkobatrachus_polonicus
???0?0???10??????????1???????010??1
?00???????????????????
Prosalirus_bitis ???????1??00???11?????????1??????0?0????0??0???0?0???10{1
2}???0???1?0???1?????01??1?20?1??0?????????
Vieraella_herbsti 1?0?01?1011001???001??1001101101?010?????00??1?00?{1
2}?0?0?0?0?0????0?001?0???01??1??0?1?0??????????
Notobatrachus_degiustoi 000000?101{1
2}?1100100011100l?001010000?1?00000?2?0021?0101000{1
2}0??1000001?0??0010?1020?1??0?????????
Ascaphus_truei
00111?010220?10?01?111100100?0?1011100001000?2001220010200000001011001?0
020111010?00111101?000000
Leiopelma_hochstetteri
00111?010220?20?01?1111001001201011101001000?2001220010210000001000001?0
030111010200111101?100000
Alytes_obstetricans
00001?010210?21?10?120100110120111110001000?30110200112?10?010101100001
031111011200120101?1000?0
Barbourula_busuangensis
0000000100100210000120100110120111110010000?301002?0112?20?0001011?10?1
031111010100100101?1000?0
Bombina_orientalis
0000010102101210100130100110120111110000100003010020011 2?2000001011100?1
031111010{0 1}00100101?1000?0
Discoglossus_pictus 000000010{1
2}1012101001201101101201111100110000030100200112110?0101011100010311110l
1210120101?1000?0
Eodiscoglossus_santonjae
000001?1001001??100?201001?0????????????0?00?3010?2?01{0
1}2110?0????1??00?1????11?102?01?01?1???????
Callobatrachus_sanyanensis
000?0??10?20?2??100?201001101201????????0000?2010?2?0112110?0??1011??0?0???
111?1?2??1?01?1??0????

Mesophryne_beipiaoensis
000?0??1?????2?0000??01101?0????11?0?0?000??2020?2?01120?0????10??0010????
110?1?2?01?01?0??0?0??
Pipa_pipa 10011{0 1}{1 2}110210221011011300111???10211111201{0
1}1?31100321122132?1011001{0 1}010102?11101002110011 1?201000
Xenopus_sp {0 1}021000110201231011011300 11{1
2}???102111012010103{0
1}102321122132?1011012010010 2?111010021110111?201000
Rhinophrynus_dorsalis
100000?11021?21?0011102001101 1?10210020011110301004?1102?12??001101?01?20
0111101120010011 1?210010
Palaeobatrachus_diluvianus 10000??110?0?21?00102{0
1}10011?1??10210?0?00001?312023?0112?12????1?11??1?1??011{0
1}?1?2??1?0111???????
Neusibatrachus_wilferti 0??????11??0????1?0??0?????01??1021??0?00?0??31202{3
4}20102112?0???0111000?????1??1????1????????????
Hyla_sp {0
1}0011?010220?21?10?120101010110011 11?1020?01?312024?0112112?{0
1}1011001010001?11121010?100111?210011
Limnodynastes_peronii
10010?0101?0?21?10?120100110110011 11?1020101?312114{1
2}0102112?110110000101{0 1}1?11121021?1001{0 1}1?210011
Yizhoubatrachus_macilentus
00????0101?1???1010??0?????0????001?01?0000??2?1????0112?12?0???0111001????1
1??1????1??0????0????
Pelodytes_caucasicus
001111010010 12?100012010011012011101000100010302004111 22?2??01011001011?
1??1111111001002?1?210100
Pelodytes_punctatus
0010000102201 2?10001201001101201111000100010302014111 22?2??01010101011?
1??11?1111001002?1?210100
Pelodytes_ibericus
0011100102201 2?10001201001101201111000100010302014111 22?2??01010101011?
1??11?1111001002?1?210100
Aerugoamnis_paulus 101001010?{1 2}012?1?00120100110???1?????{0
1}??0?01?30?104111120201 0??100010??1???11??1?2001?00?{0 1}???????
Tephrodytes_brassicarvalis
010000?1001?02???001201111??????????????0?01130200411122?{1
2}2?0???1001??????111??1?10?1??2?{0 1}???????
Megophrys_nasuta
00010?0100?1?2??10?120101010110111110?020001130200410 1?2?{0
3}1?0201100?01?01??11101?1?0100111?21000?
Elkobatrachus_brocki
?0?10??10210?2?010??2?????10?????1100?????01130?0?41111212 0?0??1101001?011?
11??1???01?00?1??1????

Prospea_holoserisca 10010??102111{1
2}???0012?100110???1?11?010???01?3020?{3
4}11122021?0??1??01001?01111?01?20?1??1?22?1????
Spea_multiplicata
0001002102111 2??10112120011012111111010200 01?3020041111203{1
2}001011001001101?1110112001001122?10100
Spea_hammondii
00110?2102?112??10?121200110?211?1?1010?00011302004111120310010110010 01?0
1?11?01?200100??22?10100
Spea_intermontana
000100210121 12??101121200110121101110102000113020041111203200101100100 1?
01?1110112001001122?10100
Spea_bombifrons
001101210211 12?010112120011012111111010200011302004111120310010110010 01?
01?1110112001001122?10100
Scaphiopus_hurterii
020100210021 02??1001211111101211111101020001130200411?120??001011001001?
01?11?01?200100??21?10100
Scaphiopus_holbrookii
020100210011 02??1001211111101211111101020001130200411112032001011001001?
01?11?011200100??21?10100
Scaphiopus_couchii
020100210011 02??1011211111101211111101020001130200411112032001011001001?
01?11?01?200100??21?10100
Scaphiopus_skinneri
020111210021 02?0100121111110121111111010?0?0?13020???1112?32?0???10???0????
??????????????????????
Scaphiopus_guthriei 0210??21002102????0?211111 10?{1
2}111111011???0?1??????????????????100?????????????????????????????
Macropelobates_osborni
010????1001112??????20111110?????1??0?0?0?01?3020?4?1122022?0??11????1?????
11??112011?00?21?1????
Pelobates_decheni
010000?1001102???00?201110??????????????????130?0?41?122022?0??1???????0???1
1??1?2?01??0?????????
Pelobates_varaldii
01000?2110?102??10?120111010100111110?02000113020041112203200101100101 1?
11?11?01?200100??21?10100
Pelobates_cultripes
01000121100102?0100120111010100111110102000113020041112 20{2
3}20010110?1011011?1110112{0 1}01001?21?10100
Pelobates_fuscus 01000?211011021?100120111{0
1}101001111101?2200113020041112203200101100101101 1?111011200100{0
1}121210100

Pelobates_syriacus
01000?2110?102??10?120111010100111101?22001130200411122032001011001011?
11?11?011200100??21?10100
Gobiates_spinari
02000021021011?0??0020111110?0?111?101???0??13000?2?1112110?0??111010?????
??1??1?0?01????0???????
Eopelobates_anthracinus
00010?21001102???00?20111110?10?111101020?0?13020?4?11220{2
3}2?0??1110??1?{0 1}1??11??102??1?0000???????
Eopelobates_bayeri
01010021102102???00?201111101????????1????0??3020?4111220{2
3}200??111?1????1???1??1????1?00?{0 1}???????
Eopelobates_deani 1011100100{1
2}112?1100120110110?????????????????302{0
1}?41112201200??111?????011?11??1????1?00?0???????
Eopelobates_grandis
010111?1002102????0?211111101??????????????01?3020?4111?2?{1
2}?00??111??????11?11??1??101?0{0 1}?0???????
Genibatrachus_baoshanensis
000000010010002?01000201001101 1?011??010?0?0113021020010211000??10011000{
0 1}0?111121010?1?00?0??0????
Electrorana_limoae
101011000?2011?1010120?0?100?11011???1011?01??0??1??????????0???1?11000?00?
?1???????1??2?0???????

;

END;

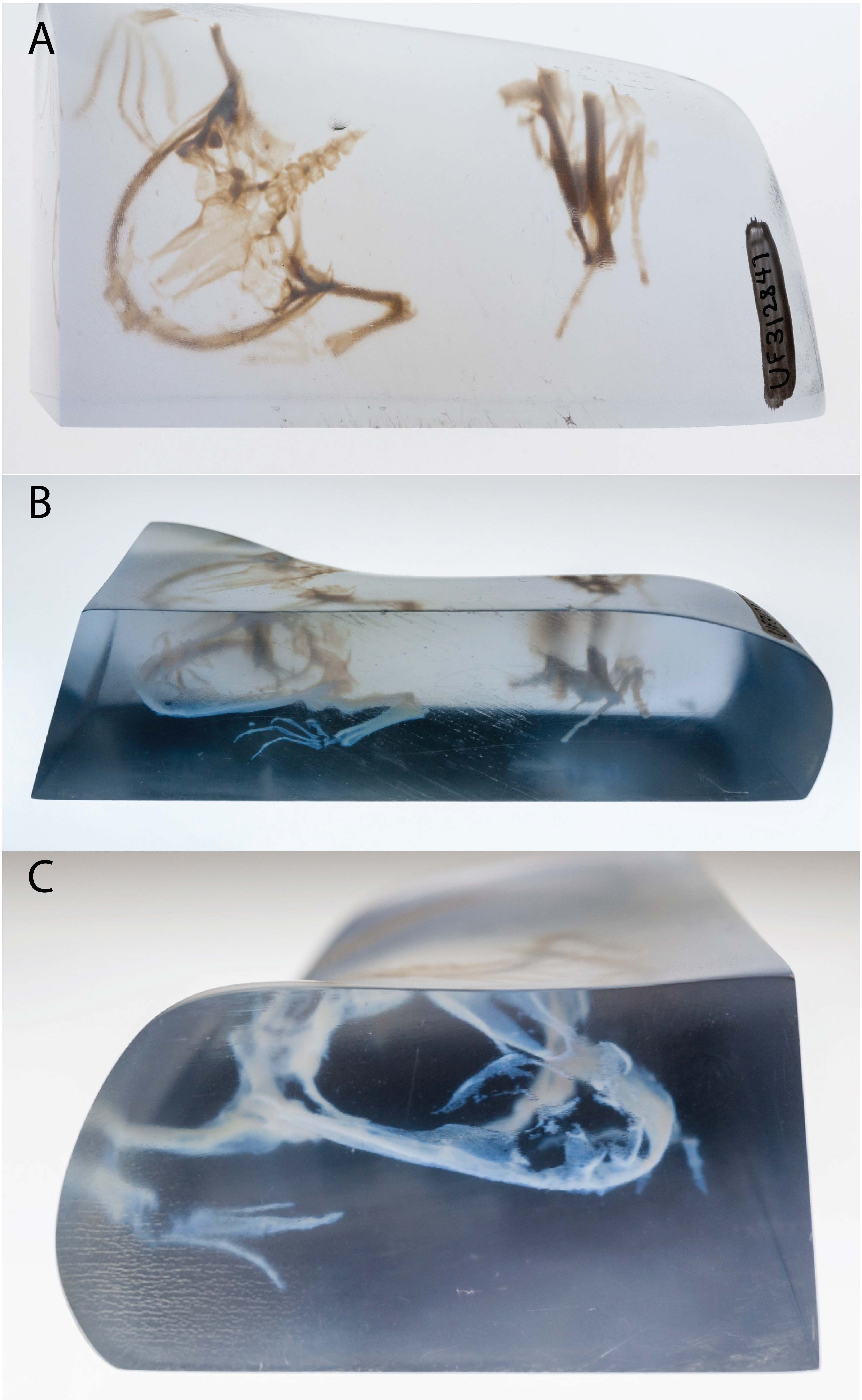
A
UF312847
B
C
